# Combined Phylogeographic Analyses and Epidemiologic Contact Tracing to Characterize Atypically Pathogenic Avian Influenza (H3N1) Epidemic, Belgium, 2019

**DOI:** 10.3201/eid2902.220765

**Published:** 2023-02

**Authors:** Steven Van Borm, Géraldine Boseret, Simon Dellicour, Mieke Steensels, Virginie Roupie, Frank Vandenbussche, Elisabeth Mathijs, Aline Vilain, Michèle Driesen, Marc Dispas, Andy W. Delcloo, Philippe Lemey, Ingeborg Mertens, Marius Gilbert, Bénédicte Lambrecht, Thierry van den Berg

**Affiliations:** Sciensano, Brussels, Belgium (S. Van Borm, G. Boseret, M. Steensels, V. Roupie, F. Vandenbussche, E. Mathijs, A. Vilain, M. Driesen, M. Dispas, B. Lambrecht, T. van den Berg);; Rega Institute KU Leuven, Leuven, Belgium (S. Dellicour, P. Lemey);; Université Libre de Bruxelles, Brussels (S. Dellicour, M. Gilbert);; Royal Meteorological Institute of Belgium, Uccle, Belgium (A.W. Delcloo);; Federal Agency for the Safety of the Food Chain, Brussels (I. Mertens)

**Keywords:** influenza, respiratory infections, viruses, avian influenza, whole-genome sequencing, molecular epidemiology, Belgium

## Abstract

The high economic impact and zoonotic potential of avian influenza call for detailed investigations of dispersal dynamics of epidemics. We integrated phylogeographic and epidemiologic analyses to investigate the dynamics of a low pathogenicity avian influenza (H3N1) epidemic that occurred in Belgium during 2019. Virus genomes from 104 clinical samples originating from 85% of affected farms were sequenced. A spatially explicit phylogeographic analysis confirmed a dominating northeast to southwest dispersal direction and a long-distance dispersal event linked to direct live animal transportation between farms. Spatiotemporal clustering, transport, and social contacts strongly correlated with the phylogeographic pattern of the epidemic. We detected only a limited association between wind direction and direction of viral lineage dispersal. Our results highlight the multifactorial nature of avian influenza epidemics and illustrate the use of genomic analyses of virus dispersal to complement epidemiologic and environmental data, improve knowledge of avian influenza epidemiologic dynamics, and enhance control strategies.

Wild birds in the orders Anseriformes and Charadriiformes are considered natural reservoirs of avian influenza viruses (AIVs; family Orthomyxoviridae), maintaining the 16 hemagglutinin (H1–16) and 9 neuraminidase (N1–9) viral subtypes circulating in bird populations (*1*). This reservoir promotes virus evolution, long-range spread, and frequent spillover events to other animal species, including poultry (*2*,*3*). Most AIVs have low pathogenicity, which is defined by intravenous inoculation of chickens 4–8 weeks of age. In contrast, some H5 and H7 subtype strains have high pathogenicity, causing systemic infection and high mortality in chickens (*4*). A polybasic motif within the endoproteolytic cleavage site of the H5 or H7 hemagglutinin precursor protein was recognized as a major determinant of high pathogenicity (*5*,*6*).

Complete sequences of AIV are increasingly used to model and trace avian influenza epidemics both locally (*7*–*12*) and globally (*13*). Moreover, analysis of genomic sequences can be integrated with epidemiologic and environmental data to improve outbreak investigations (*14*–*16*), estimate importance of different epidemiologic parameters (*17*), investigate the effects of external factors on virus dispersal (*13*), or assess the effect of implemented control measures (*18*).

In 2019, Belgium experienced an epidemic of low pathogenicity AIV of subtype H3N1 with unexpectedly high mortality and severe clinical signs in breeder and laying hens (*19*). After the initial outbreak in January and a voluntary partial depopulation of hens in the index farm, a closely related low-pathogenicity AIV was detected in April in the same index farm; a neuraminidase stalk deletion was detected in the virus, indicating viral adaptation to poultry (*19*). Subsequently, the virus spread to 81 additional farms in Belgium and 3 epidemiologically linked farms in France (*19*).

The overall goal of this study was to characterize and explain the epidemiologic dynamics of the 2019 AIV H3N1 epidemic by analyzing epidemiologic, viral genomic, geographic, and environmental data covering most affected farms. Specifically, we aimed to reconstruct the spread of the virus and test hypotheses regarding potential drivers of virus dispersal.

## Methods

### Case Definition and Epidemiologic Data Collection

**Figure 1:**
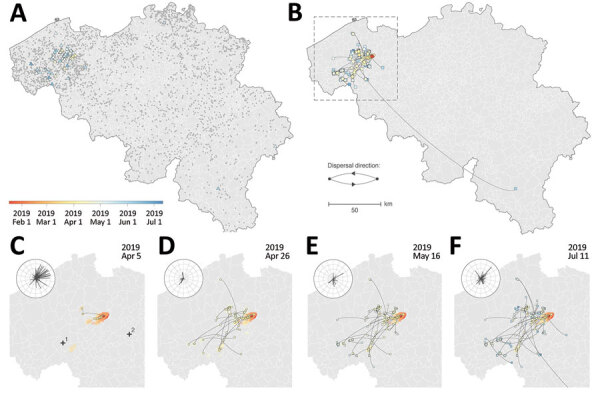
Spatiotemporal dispersal history of H3N1 lineages in study combining phylogeographic analyses and epidemiologic contact tracing to characterize the atypically pathogenic avian influenza (H3N1) epidemic in Belgium during 2019. A, B) We mapped the spatiotemporal distribution of H3N1 outbreaks (triangles) among the distribution of Belgian poultry farms (gray dots) (A) and the maximum clade credibility tree obtained by continuous phylogeographic inference on the basis of 1,000 posterior trees (B). The tree is superimposed on 80% highest posterior density polygons reflecting phylogeographic uncertainty associated with inferred positions of internal nodes. Tip (squares) and internal (circles) nodes are displayed, and dispersal direction of viral lineages is indicated by the edge curvature (anticlockwise). Outbreaks, tree nodes, and highest posterior density regions are all colored according to their date of occurrence. C–F) Four snapshots of the area shown in the box in panel B, which display the dispersal history of H3N1 lineages through time and on which we coplotted the wind direction and intensity (length of line, not used for hypothesis testing) recorded for the days in each period. The period was defined as the time between the date of the previous snapshot and the date of the snapshot under consideration. Wind direction and intensity were averaged measurements taken at 2 meteorological stations (1, Beitum; 2, Melle). A visual comparison between the time-scaled tree and the phylogeographic reconstruction is provided in Appendix.

A case or outbreak was defined as a farm with animals infected by AIV subtype H3N1, confirmed by molecular testing (Appendix). We collected data on 62 of 82 affected farms by using individual semistructured questionnaires, encoding farmer’s documents (production data, deliveries, and visitor registries), and tracing cadaver transport. We encoded all data in a harmonized format to include contact tracing, zootechnical and clinical information, and geographic location (Appendix). The extracted data enabled the assignment of samples to different production units within a farm. We analyzed contacts between farms (feed/manure/cadaver trucks, veterinarians, hatcheries, slaughterhouses, technicians, visitors) and networks between operators (Appendix Table 1). We considered the infectious period to be <7 days before and after the onset of symptoms (reported by the farmer) as validation of a probable transmission event. We separated transmission networks into transport contact networks, comprising farms connected through commercial movement of a vehicle (1 specific time on 1 specific day), and social contact networks, comprising farms linked through social connections occurring several times during the infectious period, such as family or neighbor visits. We obtained hourly and daily records of wind directions and speeds from August 1, 2018, to July 31, 2019, from 2 synoptic weather stations situated in Beitem and Melle, Belgium, that were close to the initial outbreak area (Figure 1). We detected spatiotemporal case clusters by using SaTScan version 9.6 (https://www.SaTScan.org). We used time-associated settings according to incubation and clinical periods reported by farmers that were estimated to last a total of 14 days, from infection (day –7) to recovery (day +7), and according to the entire epidemic period (15 weeks). To align to zones defined in surveillance recommendations (Directive EU/2005/94 for AI surveillance), we defined circular clusters of a 3 or 10 km radius. We mapped the identified clusters by using QGIS version 3.18 (https://qgis.org).

### AIV Whole-Genome Sequencing

We extracted viral RNA from clinical samples or virus isolates and amplified influenza A segments by reverse transcription PCR using universal primers (*20*). We performed Illumina-based (https://www.illumina.com) sequencing, aiming for a minimum of 0.5 million read pairs per sample. We performed reference-based (GenBank accession nos. MN006980–7) AIV genome assembly (Appendix). We concatenated virus genomes by joining size-sorted segment sequences (S1 through S8) without inclusion of a spacer sequence. The resulting sequenced consensus genomes (n = 103) were added to the GISAID EpiFlu database (https://www.gisaid.org), where the genome from the epidemic index case was previously submitted (*19*) (Appendix Table 2). We verified the monophyletic, single introduction nature of the outbreak by using exploratory maximum likelihood phylogenetic analysis (Appendix).

### Spatially Explicit Phylogeographic Reconstruction

We aligned the 104 concatenated H3N1 genomes (representing 70 of 82 affected farms) by using MAFFT version 7.310 (*21*) and masked regions without coverage during pairwise comparisons of genomes. For each concatenated genome, we included the geographic coordinates of the affected farm, farm and production unit identification, and sampling date of the original clinical sample in the metadata. We performed a regression of root-to-tip genetic distances against sequence sampling times to assess the phylogenetic temporal signal by using the program TempEst (*22*) (yielding a coefficient of determination R^2^ = 0.32) on the basis of a maximum likelihood tree generated with SeaView version 5.0.5 (*23*). We assessed the absence of a recombination signal by using the Φ-test (*24*) implemented in the program SplitsTree 4 (*25*).

For spatially explicit phylogeographic reconstruction of H3N1 lineage dispersal history during the epidemic, we used the relaxed random walk diffusion model (*13*,*26*,*27*) implemented in the software package BEAST 1.10 (*28*). This model enables a joint inference of time-calibrated phylogenetic trees and continuous character mapping of longitude and latitude at internal tree nodes (Appendix). We used 1,000 trees sampled from the posterior distribution for different post hoc analyses.

### Potential Drivers of Virus Spread

To investigate the effect of wind direction on H3N1 lineage dispersal, we compared wind direction data with dispersal directions of lineages inferred through our phylogeographic analysis and with dispersal directions of the same lineages in a null dispersal model (*29*). For each phylogenetic branch, for which position was inferred or randomized in the study area, we then computed the angle between dispersal direction and wind direction at the time of the dispersal event. We used a Bayesian approach (Appendix) to test the hypothesis that wind direction was correlated more with inferred than randomized dispersal direction for viral lineages (*30*,*31*). We interpreted Bayes factors (BFs) as previously described (*32*), where 3<BF<20 corresponded to positive support and BF>20 corresponded to strong support. We performed this test during different time periods (Figure 1) and with different geographic distance cutoff values (1, 2, 5, and 10 km) to select phylogenetic branches for inclusion in the analysis. The 4 time periods were delineated by key events and decisions made during the epidemic, such as key dates in human activity and behavior toward avian influenza biosecurity measures (Appendix).

We used a Bayesian approach (*29*) to assess the phylogenetic signal associated with 3 categorical epidemiologic covariates attributed to virus source farms: spatiotemporal SaTScan clusters, transport contact networks (including feed delivery, manure and cadaver collection, and live animal transport), and social networks (same veterinarian, family, or neighbors) during the epidemic. We used the same 1,000 trees sampled from the posterior distribution and the R package phytools (http://www.phytools.org) to estimate the Blomberg *K* statistic. The *K* statistic measures the phylogenetic signal of a covariate by comparing the observed signal with the signal under a Brownian motion model of trait evolution (*30*,*31*). The statistical support (BF) associated with the inferred distribution of *K* for a given covariate was evaluated by comparing with its corresponding null distribution (Appendix) (*28*,*33*). BF support levels were interpreted as previously described (*32*,*34*).

## Results

### Epidemiologic Findings

Most affected poultry farms (91.5%) were situated in a single area of dense poultry farming (Figure 1, panel A) and mainly involved laying hens (mean age at onset of symptoms was 45 weeks). All the identified spatiotemporal clusters were located in the area of dense poultry farming, including 4 clusters with a 3 km radius (Appendix Figure 1) and 3 clusters with a 10 km radius. Of the 4 clusters with a 3 km radius, cluster 1 included the index case, clusters 2 and 3 represented short distance dispersal in a westerly direction, and cluster 4 represented a medium distance (<50 km) dispersal in a southwesterly direction (Appendix Figure 1). A long-distance dispersal event (>100 km) in the southeasterly direction into the province of Luxemburg was linked with the transport of live animals from cluster 2. A potential long-distance dispersal event in the northeasterly direction consisted of 2 weak PCR-positive asymptomatic farms in the province of Antwerp; no data were obtained, excluding those farms from the phylogenetic analysis. Contact tracing data from outbreak investigation efforts covered 62 (75%) of 82 affected farms. Documented anthropogenic transmission routes (Appendix Table 1) showed potential connectivity between affected farms, involving transport (live animals, eggs, feed, manure, or cadavers) and human movements between farms. We identified 6 transport contact networks and 9 social contact networks.

### Whole-Genome Sequencing

Of the 104 virus sequences (representing 85% of the affected farms during the epidemic), 73 were complete genomic sequences (all segments had >95% coverage), 5 were near complete sequences (some segments had only partial coverage), and 26 were partial sequences (some segment sequences were missing) (Appendix Table 3). A preliminary phylogenetic investigation confirmed that all sequences corresponding to the 2019 epidemic in Belgium were clustered together within a monophyletic clade (Appendix Figure 2). A reasonable temporal signal was highlighted by our root-to-tip regression analysis (R^2^ = 0.35; Appendix Figure 3). We did not find statistical support for a recombination signal (p = 0.442).

### Phylogeographic Reconstruction

Spatially explicit phylogeographic reconstruction (Figure 1, panel B; Appendix Figure 4) confirmed that the spread of the virus began within an area near the index case. Local circulation during the initial epidemic phase was suggested by the presence of multiple internal nodes dating before the reoccurrence of clinical signs in chickens on April 5, 2019, in the same index farm (Figure 1, panel C). A relatively fast initial spread of the virus occurred in an area of dense poultry farming toward the southwest (Figure 1, panel D), followed by local short distance dispersal events in the affected area and medium-to-long distance dispersal events without a clear directional trend. In addition, we observed secondary dispersal clusters and a further extension of the affected geographic area (Figure 1, panels E, F). Our phylogeographic reconstruction also confirmed a link between the isolated long-distance (>100 km) (Figure 1, panel B) dispersal event in the province of Luxemburg and the area corresponding to spatiotemporal cluster 2 (Appendix Figure 1).

### Potential Drivers of AIV Spread

Moderate support for an association between virus dispersal direction and wind direction was only found for lineage dispersal events >5 km (BF = 3.08) and >10 km (BF = 3.76). When we analyzed different time periods (Figure 1, panels C–F; Appendix), we found positive but weak support for an association between virus dispersal and wind direction during the second time period (April 6–26, 2019) and, again, only for lineage dispersal events >5 km (BF = 3.33) and >10 km (BF = 4.05).

**Figure 2:**
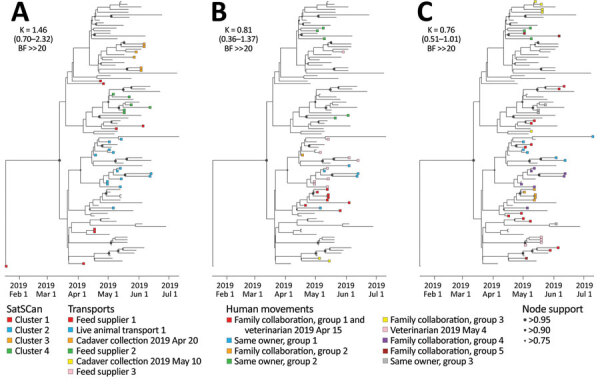
Analysis of the phylogenetic signal associated with 3 covariates in study combining phylogeographic analyses and epidemiologic contact tracing to characterize the atypically pathogenic avian influenza (H3N1) epidemic, Belgium, 2019. We assessed the phylogenetic signal associated with 3 covariates: A) Spatiotemporal SaTScan clusters (https://www.SaTScan.org); B) transport contact networks; C) social contact networks. Tree tip nodes are colored on the basis of the cluster or network to which they belong. For each covariate, we also report the estimated Blomberg *K* statistic and associated 95% highest posterior density interval (in parentheses) and BF support. BF, Bayes factor; ≫, much greater than.

We analyzed evolutionary relationships among viruses from affected farms and potential covariates: SaTScan spatiotemporal clustering, transport-related contact networks, and social contact networks. We used a Bayesian approach to assess the phylogenetic signal associated with each of these covariates (*30*) (i.e., the tendency for farms sharing genetically similar viruses to share the same covariate value). We observed strong statistical support (BF ≫ [much greater than] 20) for the phylogenetic signal associated with each tested covariate (Figure 2). In particular, the strong association between the 3 km geographic clusters identified in the SaTScan analysis and the phylogenetic reconstruction (Figure 2) illustrated the importance of geographic proximity as a main driver of H3N1 dispersal. Cases from SaTScan cluster 1 (Figure 2), which appeared first during the epidemic, were spread over the entire phylogenetic tree, while the other spatiotemporal clusters formed distinct clades within the tree, indicative of secondary spread and diversification. Both transport (including feed delivery, manure and cadaver collection, and live animal transport) and social contact (same veterinarian, family, or neighbors) networks identified through epidemiologic investigations also had strong phylogenetic signals (BF >>20 for both covariates) (Figure 2). However, contact variable mapping to the tree did not perfectly fit into unique clades, leaving several traced contacts invalidated by the phylogenetic analysis (Figure 2).

## Discussion

The atypical pathogenicity, high and prolonged viral excretory titers, and the swift dispersal observed for the low-pathogenicity H3N1 virus (*19*,*35*) affecting poultry in Belgium during 2019 merited an in depth investigation of its dispersal dynamics. For this purpose, spatially explicit phylogeographic reconstruction on the basis of AIV whole-genome sequence analysis was used to supplement and validate the available descriptive contact tracing data collected during and after the epidemic. This approach had 3 main advantages. First, sufficiently diverse genetic data enabled the reconstruction of a high resolution and objective spatiotemporal dispersal history of viral lineages. Second, available samples from routine diagnostics during the epidemic permitted high coverage of affected farms, although not necessarily of asymptomatic farms because of surveillance and methodological biases. Third, the reconstructed dispersal history of viral lineages could be used to test the validity of hypotheses formulated from epidemiologic data, thereby upgrading the contact investigation from a descriptive to qualitative assessment of potential drivers of the epidemic.

AIV whole-genome sequences provide high resolution data that permitted detailed reconstruction of the dispersal history of viral lineages (*11*,*36*). Moreover, phylogeographic analyses of whole genomes were previously used to verify or supplement epidemiologic tracing (*14*–*16*), predict AIV wildlife to poultry jumps (*10*), and associate eco-climatic host density predictors (*15*) or environmental factors (*17*) with AIV outbreak patterns.

Our spatially explicit phylogeographic reconstruction confirmed the origin of the epidemic was near the index farm. The first infection with a low-pathogenicity AIV (H3N1) occurred in outdoor laying hens at a farm in January 2019, where the farmer depopulated only the affected flocks on a voluntary basis, maintaining the healthy flocks in other production units. A closely related virus was detected on April 5, 2019 (date of official notification of an AIV H3N1 outbreak) and contained a neuraminidase stalk deletion indicative of adaptation to poultry and an alternative hemagglutinin precursor protein activation mechanism (*19*,*35*). Several internal nodes of our phylogeographic reconstruction dated before the reemergence of the adapted virus in the index farm, suggesting continued local circulation accompanied by virus diversification in or near the index farm. In the second phase, the virus spread into an area with a high density of poultry farms. Secondary spread included both short and medium distance transmission events and a single long-distance transmission event caused by direct transportation of live animals from a subclinically affected farm.

The windborne virus spread hypothesis was frequently suggested by farmers. We only found weak statistical support for effects of wind direction on virus spread during the early phase of the epidemic when uncontrolled viral spread occurred before the poultry sector increased biosecurity awareness (starting around April 26, 2019). Effects were limited to long distance (>5 km) spread. The absence of correlation between virus dispersal direction and wind direction for shorter distances seems counter-intuitive, especially when considering the dense poultry farming area where the outbreak occurred in combination with the strong correlation between spatiotemporal clustering (at distances <3 km) and genetic relatedness of viral genomes. Wind-based AIV dispersal remains a much debated topic. Strain specific excretion patterns (duration, respiratory versus intestinal, concentration), outbreak specific farm biosecurity and farm organization (number of animals, ventilation, disinfection of vehicles and fomites), and meteorological conditions have a major effect on virus survival, aerosolization, and dispersal. Some studies predicted a wind contribution of up to 20% of dispersal events for a highly pathogenic AIV epidemic (*12*,*37*,*38*), whereas other studies predicted an effect limited to very short distances of <1 km for highly pathogenic AIV (*39*) and no effect of wind dispersal for low-pathogenicity AIV (*40*). These studies illustrate the importance of additional factors such as poultry type and density, housing type, biosecurity protocols and of other anthropogenic dispersal mechanisms in the particular context of a given AIV epidemic (*41*,*42*).

Farms in epidemiologically defined contact networks had a marked tendency to host viruses more closely related within the phylogenetic tree. Those contact networks promoted efficient virus transmission. The initial spatiotemporal cluster in the epidemic source region corresponded to relatively widespread taxa within the phylogenetic tree, which highlights a pronounced genetic diversification in the index farm. Although we cannot formally exclude diversification in the surrounding source area following multiple introductions in the index farm, we believe this process is highly unlikely because all but the first virus sample contained a neuraminidase stalk deletion marker for poultry adaptation. Of note, the 3 categorical epidemiologic variables, spatiotemporal clustering, transport networks, and social contact networks, are not entirely independent. Spatial proximity or social links might, for instance, have an influence on documented transport links between farms, which was illustrated by social networks such as family group 2 that was represented as part of larger transport network, including feed supplier 1 (Figure 2). In some instances, contact networks identified through tracing efforts host various phylogenetic clades, such as family collaboration group 3, which experienced 2 independent virus introductions. Another example was family collaboration group 5, where only 2 of 3 farms contained genetically similar viruses. In addition, suspected contact networks are sometimes invalidated by the phylogenetic analysis. For instance, the feed supplier 3 contact network did not correspond to taxa directly connected within the maximum clade credibility tree. Although the association between spatiotemporal and epidemiologic contact networks and genetic reconstruction is highly supported, the examples of imperfect associations between epidemiologic and genetic reconstructions indicate that farms in those contact networks were affected by genetically diverse viruses.

Whole-genome analysis of AIV dispersal provides additional insights that can be used to evaluate control policies and enhances information obtained from descriptive epidemiologic investigations. For example, our phylogeographic reconstruction suggests unnoticed virus circulation and diversification before H3N1 reemergence in the index farm. In addition, our phylogenetic signal analyses invalidated several epidemiologically identified contact networks that did not contain genetically related viruses. Finally, our hypothesis testing confirmed that, in addition to spatiotemporal proximity, transport and social contact variables were likely the main factors involved in virus spread during both the initial phase and secondary cluster establishment.

Beyond showing the highly complementary nature of epidemiologic contact tracing and genomic dissemination reconstruction, our findings highlight the importance of surveillance and swift implementation of biosecurity measures. Enhanced surveillance could improve the likelihood of detecting cryptic virus circulation, diversification, and adaptation, and would also enable more rapid implementation of outbreak intervention measures. In addition, enhanced surveillance could improve the coverage of both epidemiologic and genetic data, ultimately improving our understanding of epidemic dispersal dynamics and providing novel insights for surveillance design and outbreak management strategies.

Appendix Additional information for combining phylogeographic analyses and epidemiologic contact tracing to characterize atypically pathogenic H3N1 avian influenza epidemic, Belgium, 2019.
