# Supplementary material for: Combined Phylogeographic Analyses and Epidemiologic Contact Tracing to Characterize Atypically Pathogenic Avian Influenza (H3N1) Epidemic, Belgium, 2019
Source: Emerg Infect Dis. 2023 Feb;29(2):351–9. doi: 10.3201/eid2902.220765 (PMC9881769; doi:10.3201/eid2902.220765)
Supplement: Appendix — Additional information for combining phylogeographic analyses and epidemiologic contact tracing to characterize atypically pathogenic H3N1 avian influenza epidemic, Belgium, 2019. [file 22-0765-Techapp-s1.pdf]

Article DOI: https://doi.org/10.3201/eid2902.220765

Combined Phylogeographic Analyses and Epidemiologic Contact Tracing to Characterize Atypically Pathogenic Avian Influenza (H3N1) Epidemic, Belgium, 2019

Appendix

Additional Methods

Case Definition by Virological Testing

A case (outbreak) was defined as a farm that had animals infected with avian influenza virus (AIV) subtype H3N1, confirmed by virological testing. Swab and organ samples were received from the field or collected from cadavers and submitted for analysis. We performed sample pretreatment, virus RNA extraction, and AIV detection by real-time reverse transcription PCR (RT-PCR) targeting conserved influenza A matrix gene sequences (*1*) and specific H3 and N1 subtype detection (*2*,*3*) as previously described (*1–3*). We isolated viruses from AIV-positive samples by inoculating specific pathogen-free day 9 embryonated chicken eggs and passaging after 5 days by using standard procedures (*4*).

Collection of Epidemiologic Data

We collected data on 62 of 82 affected farms by using individual semi-structured questionnaires about disease emergence (date of symptom onset and symptomatology) and consecutively adopted biosecurity measures at each farm. We encoded additional information from pictures, production sheets, and handwritten documents in a harmonized format. Cadaver transport records including truck travel sheets were provided by the animal cadaver collection company (Rendac, https://www.rendac.com). We included zootechnical information (animal species, production type, daily mortality, food and water intake, circadian light cycles, weight, and age), clinical features (associated with onset date), and contact tracing information (farm visits by veterinarians, family links, feed delivery, eggs, cadavers and manure collection, and

slaughterhouse and hatchery links). We extracted identification, geographic localization coordinates (longitude and latitude), and animal registration data (including transport of live animals) from the national livestock sanitary database SANITEL (https://prd.sanitel.be; Sanitel.Net–PRD 21.6.5.0 © 2007 FAVV/AFSCA, accession date: Aug 31, 2020). The extracted information enabled assignment of samples to different production units or barns within a farm. We analyzed common professional contacts between farms (feed/manure/cadaver trucks, veterinarians, hatcheries, slaughterhouses, technicians) and constructed professional networks between operators. We considered potential transmission networks accountable when animals, transport vehicles, or visitors went from an infectious to a susceptible farm on the same day, within an infectious period of ≤7 days before and after symptom onset (determined by the farmer). We separated the identified plausible transmission networks into 2 categories: transport contact networks, comprising farms connected through commercial movement of a vehicle (1 specific time on 1 specific day); and social contact networks, comprising farms linked through social connections occurring several times during the infectious period (such as family or neighbor visits). We analyzed hourly and daily records of wind directions and speeds from August 1, 2018, through July 31, 2019, from two synoptic weather stations situated close to the outbreak areas in Beitem and Melle, Belgium.

AIV Whole-Genome Sequencing

We extracted virus RNA from clinical samples (either swabs suspended in medium or 10% wt/vol homogenized tissue samples or pooled tissues) or virus isolates by using the Macherey-Nagel Nucleospin RNA virus kit (https://www.mn-net.com) and 4 µL of GenElute-LPA synthetic carrier (Sigma, https://www.sigmaaldrich.com) instead of the kit-supplied polyA carrier RNA. We performed real-time quantitative RT-PCR of the influenza virus matrix gene to verify viral RNA yield. We amplified cDNA of all influenza A segments simultaneously by using 1 pair of influenza-specific primers that anneal to the conserved 3′ and 5′ segment ends (*5*, with modifications): MTBuni-12DEG (5′-ACGCGTGATCAGCRAAAGCAGG-3′) and MTBuni-13 (5′-ACGCGTGATCAGTAGAAACA AGG-3′). We performed RT-PCR with each primer at a final concentration of 0.2 mM, 5 µL of RNA, and Invitrogen Superscript III One-Step RT-PCR System with Platinum Taq DNA Polymerase (Thermo Fisher Scientific, https://www.thermofisher.com). We denatured viral RNA plus primers for 2 min at 95°C, cooled on ice, and then added Superscript III One-Step RT-PCR reagents according to the

manufacturer's instructions in a final reaction volume of 50 µL. PCR cycling conditions were: initial primary reverse transcription step of 60 min at 55°C; then denaturation at 94°C for 2 min; followed by 5 cycles of 94°C for 30 s, 45°C for 30 s, and 68°C for 4 min; an additional 31 cycles of 94°C for 30 s, 57°C for 30 s, and 68°C for 4 min; and a final elongation step at 68°C for 5 min. We visualized amplicon length on a 1% agarose gel. We purified RT-PCR amplicons by using AMPure XP Magnetic Beads (Beckman Coulter, https://www.beckmancoulter.com) in a ratio of 0.65 sample volume to bead volume and determined concentration fluorometrically by using a QuantiFluor® dsDNA System on a Quantus Fluorometer (Promega, https://www.promega.com).

We generated sequencing libraries from 1 ng of influenza A amplicons by using the Nextera XT kit (Illumina, https://www.illumina.com) and standard Nextera XT indices. We quantified the libraries by using a KAPA Library Quantification Kit (Roche Diagnostics, https://www.roche.com) and then pooled them equimolarly. We sequenced the libraries by using MiSeq Reagent Kit v3 (Illumina) with 2 × 300-bp paired-end sequencing according to the manufacturer's instructions, aiming for ≥0.5 million read pairs per sample.

We trimmed demultiplexed *.fastq next generation sequencing reads by using Trimmomatic v0.38 (*6*) to remove adaptor sequences and low quality bases (using the ILLUMINACLIP 2:30:10, SLIDING WINDOW:4:20, and MINLEN:50 settings). Only paired reads were retained for further analysis. We mapped quality trimmed data to GenBank reference sequences (accession nos. MN006980–7) that included 8 segments of the epidemic index case (*7*) by using Bowtie2 v2.3.4.3 (using –very-sensitive-local, -I 100 -X 750 –no-mixed –no-discordant settings; https://bowtie-bio.sourceforge.net/bowtie2/index.shtml). Reads with a minimal clip length of 5 were removed by using SamJdk v966d3dfb7 (http://lindenb.github.io/jvarkit/SamJdk.html). Nucleotide variants were called using the GATK Best Practices pipeline v4.1.3.0 (https://github.com/broadinstitute/gatk).

Preliminary Phylogenetic Analysis of Hemagglutinin Segments

To determine whether the 2019 outbreak originated from a single introduction event in the study area, we performed a preliminary maximum likelihood phylogenetic analysis to assess the monophyletic status of the clade that included all sequences from Belgium. We used IQ-TREE 2.0.3 (*8*) and a GTR (general time-reversible) model of nucleotide substitution with

empirical base frequencies and 4 free site rate categories and performed 200 bootstrap calculations to assess internal branch support. The analysis was based on the hemagglutinin gene segments of all sequences from Belgium generated in the present study and 80 H3Nx hemagglutinin segments from outside of Belgium that were selected to represent diversity of H3Nx viruses circulating in Eurasia before the 2019 H3N1 virus introduction in Belgium.

Spatially-Explicit Phylogeographic Reconstruction

We aligned the 104 concatenated H3N1 virus genomes by using MAFFT v7.310 (*9*). Regions without coverage were masked. For each concatenated genome, we included geographic coordinates of the affected farm, farm and production unit identification, and the sampling date of the original clinical sample used in the metadata. We assessed the phylogenetic temporal signal by performing a regression of root-to-tip genetic distances against sequence sampling times by using the program TempEst (*10*) ($R^2 = 0.32$) and a maximum likelihood tree generated by using the program SeaView v5.0.5 (*11*)). We assessed the absence of a signal for recombination by using the Φ-test (*12*) implemented in the program SplitsTree 4 (*13*).

For the spatially-explicit phylogeographic reconstruction of H3N1 lineages during the epidemic in Belgium, we used the relaxed random walk diffusion model (*14–16*) implemented in the software package BEAST 1.10 (*17*). This model enables a joint inference of time-calibrated phylogenic trees and a continuous character mapping of the longitude and latitude at the internal nodes of the trees. We specified a GTR+Γ substitution model, lognormal relaxed molecular clock model, skygrid coalescent tree prior, and relaxed random walk diffusion model with a gamma distribution to model the among-branch heterogeneity in dispersal velocity. Because the continuous diffusion model does not permit analysis of samples associated with exact same sampling coordinates, we added a 2 km jitter window to tips sharing identical sampling coordinates. The Markov chain Monte-Carlo algorithm was run for 10^9 generations and parameters were logged every 10^5 generations. After verifying that the estimated sampling size values were all >200, we identified and annotated the maximum clade credibility tree (MCC) by using TreeAnnotator 1.10 after having discarded 10% of sampled trees as burn-in. We used the "seraphim" R package (*18*,*19*) to extract spatiotemporal information embedded in 1,000 posterior trees and to exploit those extractions to estimate the evolution of the weighted lineage dispersal velocity through time and visualize the inferred dispersal history of H3N1 lineages. The

same 1,000 trees sampled from the posterior distribution were used for different post hoc analyses.

We performed an exploratory phylogenetic analysis to remove sequences so that monophyletic clusters of sequences sampled from the same farm were represented only by a single sequence. Those monophyletic clusters largely represent within-farm dispersal, which is characterized by noise because of the jitter used to differentiate the geographic coordinates associated with sequences from the same sampling location (*20*). The exploratory analysis was also performed in BEAST 1.10 by using the same substitution, molecular clock, and coalescent models outlined previously. Using this procedure, only 3 sequences were discarded, and the final dataset included 101 sequences.

Investigating Potential Drivers of Virus Spread

To investigate the effect of wind direction on H3N1 lineage dispersal, we compared wind direction data with dispersal directions of lineages inferred through our phylogeographic analysis and with dispersal direction of the same lineages in a null dispersal model. The null dispersal model was obtained by randomizing the geographic position of phylogenetic branches while conserving tree topology (connections among branches) and position inferred for the most ancestral node of the tree. Randomization within the study area was constrained, which was defined by the minimum convex hull polygon encompassing the position of internal and tip nodes from the 1,000 trees sampled from the posterior distribution (*19*). For each phylogenetic branch (whose position was inferred or randomized in the study area), we then computed the angle between the dispersal direction and wind direction corresponding to the time window of the considered branch. For a specific branch, wind direction was obtained by averaging daily wind directions recorded at two meteorological stations located within the study area (Figure 1, main text) that corresponded to the time window of the considered branch. For each tree, we computed the mean angle A between lineage and wind direction. Each inferred A value (A_{inferred}) was then compared with its corresponding randomized value ($A_{\text{randomized}}$) by approximating Bayes factor (BF) support as follows: BF = $[p_A/(1\text{-}p_A)]/[0.5/(1–0.5)]$, where p_A is the posterior probability that $A_{\text{randomized}}$ is $>A_{\text{inferred}}$ in samples from the posterior distribution (i.e., the frequency at which $\text{A}_{\text{randomised}}$ is $>\text{A}_{\text{inferred}}$ in the samples from the posterior distribution). The prior odds was 1 because we assumed an equal prior expectation for $\text{A}_{\text{inferred}}$ and $A_{\text{randomized}}$ (*21*,*22*). We tested the hypothesis that wind direction had greater correlation with inferred than

randomized dispersal direction for viral lineages. BF support levels were interpreted as previously described (*23*); a BF of 3<BF<20 indicated positive support, and a BF>20 or BF>>20 was strong support. We performed this test using different time periods (Figure 2, main text) and different geographic distance cutoff values (1, 2, 5, and 10 km) to determine which phylogenetic branches to include in the analysis. The 4 time periods were delineated by key events and decisions made during the epidemic, including key dates of human activity and behavior toward avian influenza biosecurity measures. The end of period 1 (August 1, 2018, through April 5, 2019) was defined by the onset of symptoms when the virus reemerged at the index farm on April 5, 2019. The end of period 2 (April 6, 2019, through April 26, 2019) was defined by the increased attention of field operators (transporters, farmers, veterinarians, feed transport, and rendering activity) to biosecurity that began on April 26, 2019, because of increased awareness of the H3N1 epidemic. The end of period 3 (April 27, 2019, through May 16, 2019) was defined by the implementation of the first official measures through ministerial decree on May 16, 2019, emphasizing reinforced passive surveillance, cleaning and disinfection of all vehicles entering or leaving a farm, one-on-one transport of poultry, restricted access to poultry farms and hatcheries by staff, farm veterinarians, or authorities' delegates, and disinfection of manure. The end of period 4 (May 17, 2019, through July 11, 2019) was defined by the last date a virus-positive sample was detected on July 11, 2019.

We used a Bayesian approach (*24*) to assess the phylogenetic signal associated with 3 categorical epidemiologic covariates attributed to virus source farms during the epidemic: spatiotemporal clusters determined by SaTScan software (https://www.SaTScan.org), transport contact networks (including feed delivery, manure and cadaver collection, and live animal transport) and social contact networks (same veterinarian, family, or neighbors). We used the 1,000 trees sampled from the posterior distribution and phytools from the R software package (The R Project for Statistical Computing, https://www.r-project.org) to estimate the Blomberg *K* statistic. The *K* statistic measures the phylogenetic signal of a covariate by comparing the observed signal in this covariate to the signal under a Brownian motion model of trait evolution on a phylogeny (*25*,*26*). For each covariate and tree sampled from the posterior distribution, we estimated 2 *K* values: 1 value derived from original covariate values that produced K_{inferred} and the other value derived from covariate values permuted among tips that produced K_{null}. Of note, permutations of covariate values were only performed among tips for which a covariate value

was initially available. The statistical support associated with $K_{inferred}$ distribution was evaluated by comparing with its corresponding K_{null} distribution and formalized by approximating a BF value. Specifically, the BF support associated with K was approximated by the posterior odds that $K_{inferred}$ was $>K_{null}$ divided by the equivalent prior probability odds (the prior probability for $K_{inferred}>K_{null}$ was considered to be 0.5): BF = $[p_K/(1-p_K)]/[0.5/(1–0.5)]$, where p_K is the posterior probability that $K_{inferred}$ was $>K_{null}$ in the samples from the posterior distribution. The prior odds was 1 because we assumed an equal prior expectation for $K_{inferred}$ and K_{null} (*21*,*22*). BF support levels were interpreted as previously described (*27*).

References

1. Van Borm S, Steensels M, Ferreira HL, Boschmans M, De Vriese J, Lambrecht B, et al. A universal avian endogenous real-time reverse transcriptase-polymerase chain reaction control and its application to avian influenza diagnosis and quantification. Avian Dis. 2007;51:213–20. PubMed https://doi.org/10.1637/7552-033106R.1

2. Payungporn S, Chutinimitkul S, Chaisingh A, Damrongwantanapokin S, Buranathai C, Amonsin A, et al. Single step multiplex real-time RT-PCR for H5N1 influenza A virus detection. J Virol Methods. 2006;131:143–7. PubMed https://doi.org/10.1016/j.jviromet.2005.08.004

3. Hoffmann B, Hoffmann D, Henritzi D, Beer M, Harder TC. Riems influenza a typing array (RITA): an RT-qPCR–based low density array for subtyping avian and mammalian influenza a viruses. Sci Rep. 2016;6:27211. PubMed https://doi.org/10.1038/srep27211

4. World Organisation for Animal Health. Manual of diagnostic tests and vaccines for terrestrial animals. Avian influenza (including infection with high pathogenicity avian influenza viruses). 2021 [cited 2022 Mar 15]. https://www.oie.int/en/what-we-do/standards/codes-and-manuals/terrestrial-manual-online-access

5. Hoffmann E, Stech J, Guan Y, Webster RG, Perez DR. Universal primer set for the full-length amplification of all influenza A viruses. Arch Virol. 2001;146:2275–89. PubMed https://doi.org/10.1007/s007050170002

6. Bolger AM, Lohse M, Usadel B. Trimmomatic: a flexible trimmer for Illumina sequence data. Bioinformatics. 2014;30:2114–20. PubMed https://doi.org/10.1093/bioinformatics/btu170

7. Steensels M, Gelaude P, Van Borm S, Van Den Berg T, Cargnel M, Roupie V, et al. Atypical pathogenicity of avian influenza (H3N1) virus involved in outbreak, Belgium, 2019. Emerg Infect Dis. 2020;26:1899–903. PubMed https://doi.org/10.3201/eid2608.191338

8. Minh BQ, Schmidt HA, Chernomor O, Schrempf D, Woodhams MD, von Haeseler A, et al. IQ-TREE 2: new models and efficient methods for phylogenetic inference in the genomic era. Mol Biol Evol. 2020;37:1530–4. PubMed https://doi.org/10.1093/molbev/msaa015

9. Katoh K, Standley DM. MAFFT multiple sequence alignment software version 7: improvements in performance and usability. Mol Biol Evol. 2013;30:772–80. PubMed https://doi.org/10.1093/molbev/mst010

10. Rambaut A, Lam TT, Max Carvalho L, Pybus OG. Exploring the temporal structure of heterochronous sequences using TempEst (formerly Path-O-Gen). Virus Evol. 2016;2:vew007. PubMed https://doi.org/10.1093/ve/vew007

11. Gouy M, Guindon S, Gascuel O. SeaView version 4: a multiplatform graphical user interface for sequence alignment and phylogenetic tree building. Mol Biol Evol. 2010;27:221–4. PubMed https://doi.org/10.1093/molbev/msp259

12. Bruen TC, Philippe H, Bryant D. A simple and robust statistical test for detecting the presence of recombination. Genetics. 2006;172:2665–81. PubMed https://doi.org/10.1534/genetics.105.048975

13. Huson DH. SplitsTree: analyzing and visualizing evolutionary data. Bioinformatics. 1998;14:68–73. PubMed https://doi.org/10.1093/bioinformatics/14.1.68

14. Dellicour S, Lemey P, Artois J, Lam TT, Fusaro A, Monne I, et al. Incorporating heterogeneous sampling probabilities in continuous phylogeographic inference—application to H5N1 spread in the Mekong region. Bioinformatics. 2020;36:2098–104. PubMed https://doi.org/10.1093/bioinformatics/btz882

15. Lemey P, Rambaut A, Welch JJ, Suchard MA. Phylogeography takes a relaxed random walk in continuous space and time. Mol Biol Evol. 2010;27:1877–85. PubMed https://doi.org/10.1093/molbev/msq067

16. Pybus OG, Suchard MA, Lemey P, Bernardin FJ, Rambaut A, Crawford FW, et al. Unifying the spatial epidemiology and molecular evolution of emerging epidemics. Proc Natl Acad Sci USA. 2012;109:15066–71. PubMed https://doi.org/10.1073/pnas.1206598109

17. Suchard MA, Lemey P, Baele G, Ayres DL, Drummond AJ, Rambaut A. Bayesian phylogenetic and phylodynamic data integration using BEAST 1.10. Virus Evol. 2018;4:vey016. PubMed https://doi.org/10.1093/ve/vey016

18. Dellicour S, Rose R, Faria NR, Lemey P, Pybus OG. SERAPHIM: studying environmental rasters and phylogenetically informed movements. Bioinformatics. 2016;32:3204–6. PubMed https://doi.org/10.1093/bioinformatics/btw384

19. Dellicour S, Rose R, Pybus OG. Explaining the geographic spread of emerging epidemics: a framework for comparing viral phylogenies and environmental landscape data. BMC Bioinformatics. 2016;17:82. PubMed https://doi.org/10.1186/s12859-016-0924-x

20. Dellicour S, Baele G, Dudas G, Faria NR, Pybus OG, Suchard MA, et al. Phylodynamic assessment of intervention strategies for the West African Ebola virus outbreak. Nat Commun. 2018;9:2222. PubMed https://doi.org/10.1038/s41467-018-03763-2

21. Suchard MA, Weiss RE, Sinsheimer JS. Models for estimating bayes factors with applications to phylogeny and tests of monophyly. Biometrics. 2005;61:665–73. PubMed https://doi.org/10.1111/j.1541-0420.2005.00352.x

22. Dellicour S, Rose R, Faria NR, Vieira LFP, Bourhy H, Gilbert M, et al. Using viral gene sequences to compare and explain the heterogeneous spatial dynamics of virus epidemics. Mol Biol Evol. 2017;34:2563–71. PubMed https://doi.org/10.1093/molbev/msx176

23. Kass RE, Raftery AE. Bayes factors. J Am Stat Assoc. 1995;90:773–95. https://doi.org/10.1080/01621459.1995.10476572

24. Martinet B, Dellicour S, Ghisbain G, Przybyla K, Zambra E, Lecocq T, et al. Global effects of extreme temperatures on wild bumblebees. Conserv Biol. 2021;35:1507–18. PubMed https://doi.org/10.1111/cobi.13685

25. Blomberg SP, Garland T Jr, Ives AR. Testing for phylogenetic signal in comparative data: behavioral traits are more labile. Evolution. 2003;57:717–45. PubMed https://doi.org/10.1111/j.0014-3820.2003.tb00285.x

26. Revell LJ. A comment on the use of stochastic character maps to estimate evolutionary rate variation in a continuously valued trait. Syst Biol. 2013;62:339–45. PubMed https://doi.org/10.1093/sysbio/sys084

27. Lee MD, Wagenmakers E-J. Bayesian cognitive modeling: a practical course. Cambridge: Cambridge University Press; 2014.

Appendix Table 1. Farm-to-farm contact tracing in study combining phylogeographic analyses and epidemiologic contact tracing to characterize atypically pathogenic H3N1 avian influenza epidemic, Belgium, 2019*

Contact type	Definition	Hypothesis	No. contacts	Remarks
Transport				
Live animals	Movement of live animals between farms during the AIV infectious period	Poultry were infected during transport or by introducing infected animals to the flock.	1	(Hatching) eggs not included in the definition of live animals
Feed	Feed delivery to different poultry facilities on the same day	Virus introduction was through transport trucks without proper cleaning and disinfection (including wheels, driver’s boots, equipment) that visited an infected farm on a given day.	8	Starting on April 24, 2019, members of the Belgian Feed Association were encouraged to apply a higher level of biosecurity
Manure	Collection of manure in ≥1 farm and delivery to the manure processing unit		0	Usually only 1 farm visited per day
Cadavers	Collection of cadavers in several poultry farms on the same day and the same itinerary		4	Beginning on April 23, 2019, affected facilities were visited at the end of the day
Social networks				
Veterinarians	Visited 2 different farms on the same day	People visited a susceptible flock after visiting an infected flock, and their vehicles and equipment potentially acted as mechanical vectors to spread AIV	8	NA
Same owner	>1 farm owned by the same person		7	NA
Family	Different farms owned by relatives known to interact through visits, animals, or feed		16	NA

*AIV infectious period was defined as ≤7 days before and after the onset of symptoms reported by the farmer. AIV, avian influenza virus; NA, not applicable.

Appendix Table 2. Sample identification, metadata, and sequence accession numbers of sequenced avian influenza virus genomes in study combining phylogeographic analyses and epidemiologic contact tracing to characterize atypically pathogenic H3N1 avian influenza epidemic, Belgium, 2019*

Accession nos.†	SeqID‡	Strain name	Outbreak no.	Province	Production unit	Production type	Sample date (dd/mm/yyyy)	Sample type
MN006980.1–MN006987.1	1–1-iPTL	A/Gallus gallus/Belgium/3497_0001/2019(H3N1)	1	WVL	1	Layers-outdoor	6/04/2019	Isolate (pooled lung + trachea)
EPI_ISL_3914869	0–1-PTL	A/Gallus gallus/Belgium/609_0001/2019(H3N1)	0	WVL	1	Layers-outdoor	18/01/2019	Pooled lung + trachea
EPI_ISL_3914870	2–1-iPTL	A/Gallus gallus/Belgium/3912_0001/2019(H3N1)	2	WVL	1	Breeders-broiler	17/04/2019	Isolate (pooled lung + trachea)
EPI_ISL_3914871	2–1-PTL-2	A/Gallus gallus/Belgium/3945_0002/2019(H3N1)	2	WVL	1	Breeders-broiler	17/04/2019	Pooled lung + trachea
EPI_ISL_3914872	3–1-PTS	A/Gallus gallus/Belgium/4396_0001/2019(H3N1)	3	WVL	3	Breeders-broiler	26/04/2019	Pool of 5 tracheal swabs maximum
EPI_ISL_3914873	4–1-iPTL	A/Gallus gallus/Belgium/3953/2019(H3N1)	4	WVL	1	Breeders-broiler	17/04/2019	Isolate (pooled lung + trachea)
EPI_ISL_3914874	4–1-PTL-2	A/Gallus gallus/Belgium/4325_0001/2019(H3N1)	4	WVL	1	Breeders-broiler	30/04/2019	Pooled lung + trachea
EPI_ISL_3914875	5–1-PCS	A/Gallus gallus/Belgium/3978_0001/2019(H3N1)	5	WVL	1	Layers-outdoor	18/04/2019	Pool of 5 cloacal swabs maximum
EPI_ISL_3914876	6–1-iPTL	A/Gallus gallus/Belgium/4010/2019(H3N1)	6	WVL	1	Layers-outdoor	23/04/2019	Isolate (pooled lung + trachea)
EPI_ISL_3914877	7–1-iBOW	A/Gallus gallus/Belgium/4008/2019(H3N1)	7	WVL	1	Breeders-broiler	21/04/2019	Intestine
EPI_ISL_3914878	8–1-PCS	A/Gallus gallus/Belgium/4070_0001/2019(H3N1)	8	WVL	1	Layers	22/04/2019	Pool of 5 cloacal swabs maximum
EPI_ISL_3914879	8–3-PCS	A/Gallus gallus/Belgium/4581_0002/2019(H3N1)	8	WVL	3	Layers	7/05/2019	Pool of 5 cloacal swabs maximum
EPI_ISL_3914880	9–2-BOW	A/Gallus gallus/Belgium/4226_0001/2019(H3N1)	9	WVL	2	Breeders-broiler	26/04/2019	Intestine
EPI_ISL_3914881	11–1-PLT	A/Gallus gallus/Belgium/4327_0001/2019(H3N1)	11	WVL	1	Layers-outdoor	30/04/2019	Pooled lung + trachea
EPI_ISL_3914882	11–1-POO	A/Gallus gallus/Belgium/4849_0001/2019(H3N1)	11	WVL	1	Layers-outdoor	13/05/2019	Pooled lung + trachea + intestine + brain
EPI_ISL_3914883	11–4-POO	A/Gallus gallus/Belgium/4849_0004/2019(H3N1)	11	WVL	4	Layers-outdoor	13/05/2019	Pooled lung + trachea + intestine + brain
EPI_ISL_3914884	12–1-iBOW	A/Gallus gallus/Belgium/4328_0005/2019(H3N1)	12	WVL	1	Breeders-broiler	30/04/2019	Intestine
EPI_ISL_3915374	13–4-iBOW	A/Gallus gallus/Belgium/4395_007/2019(H3N1)	13	WVL	4	Layers-outdoor	2/05/2019	Intestine
EPI_ISL_3914885	13–5-BOW	A/Gallus gallus/Belgium/4395_009/2019(H3N1)	13	WVL	5	Layers-outdoor	2/05/2019	Intestine
EPI_ISL_3914886	14–1-PTL	A/Gallus gallus/Belgium/4393_0001/2019(H3N1)	14	WVL	1	Layers-outdoor	2/05/2019	Pooled lung + trachea
EPI_ISL_3914887	14–1-iBOW	A/Gallus gallus/Belgium/4393_0002/2019(H3N1)	14	WVL	1	Layers-outdoor	2/05/2019	Isolate (intestine)
EPI_ISL_3914888	14–3-PTS-1	A/Gallus gallus/Belgium/4491_0005/2019(H3N1)	14	WVL	3	Layers-outdoor	3/05/2019	Pool of 5 tracheal swabs maximum
EPI_ISL_3915373	14–3-PTS-2	A/Gallus gallus/Belgium/4491_0006/2019(H3N1)	14	WVL	3	Layers-outdoor	3/05/2019	Pool of 5 tracheal swabs maximum

Accession nos.†	SeqID‡	Strain name	Outbreak no.	Province	Production unit	Production type	Sample date (dd/mm/yyyy)	Sample type
EPI_ISL_3914889	15–1-iBRA	A/Gallus gallus/Belgium/4452_0002/2019(H3N1)	15	WVL	1	Breeders-broiler	3/05/2019	Isolate (brain)
EPI_ISL_3914890	16–1-POO	A/Turkey/Belgium/4771_0001/2019(H3N1)	16	WVL	1	Broiler-turkey	10/05/2019	Pooled lung + trachea + intestine + brain
EPI_ISL_3914891	16–1-BOW	A/Turkey/Belgium/4453_0002/2019(H3N1)	16	WVL	1	Broiler-turkey	3/05/2019	Intestine
EPI_ISL_3914892	17–1-POO	A/Turkey/Belgium/4712_0001/2019(H3N1)	17	WVL	1	Broiler-turkey	9/05/2019	Pooled lung + trachea + intestine + brain
EPI_ISL_3914893	19–2-PCS	A/Gallus gallus/Belgium/4735_0002/2019(H3N1)	19	WVL	2	Breeders-broiler	9/05/2019	Pool of 5 cloacal swabs maximum
EPI_ISL_3914894	19–1-PCS	A/Gallus gallus/Belgium/4428_0001/2019(H3N1)	19	WVL	1	Breeders-broiler	2/05/2019	Pool of 5 cloacal swabs maximum
EPI_ISL_3914895	20–1-PCS	A/Gallus gallus/Belgium/4464_0001/2019(H3N1)	20	WVL	1	Breeders-broiler	3/05/2019	Pool of 5 cloacal swabs maximum
EPI_ISL_3914896	21–3-PTL	A/Gallus gallus/Belgium/4468_0006/2019(H3N1)	21	WVL	3	Breeders-broiler	3/05/2019	Pooled lung + trachea
EPI_ISL_3914897	21–1-POO	A/Gallus gallus/Belgium/4888_0001/2019(H3N1)	21	WVL	1	Breeders-broiler	14/05/2019	Pooled lung + trachea + intestine + brain + oviduct
EPI_ISL_3914898	21–2-POO	A/Gallus gallus/Belgium/4888_0002/2019(H3N1)	21	WVL	2	Breeders-broiler	14/05/2019	Pooled lung + trachea + intestine + brain + oviduct
EPI_ISL_3914899	22–6-PCS	A/Turkey/Belgium/4462_0001/2019(H3N1)	22	WVL	6	Breeders-broiler-turkey	3/05/2019	Pool of 5 cloacal swabs maximum
EPI_ISL_3914900	22–4-PTS	A/Turkey/Belgium/5953_0003/2019(H3N1)	22	WVL	4	Breeders-broiler-turkey	7/06/2019	Pool of 5 tracheal swabs maximum
EPI_ISL_3915375	23–2-PCS	A/Gallus gallus/Belgium/4766_0002/2019(H3N1)	23	WVL	2	Breeders-broiler	10/05/2019	Pool of max 5 cloacal swabs
EPI_ISL_3914901	23–1-PCS	A/Gallus gallus/Belgium/4767_0002/2019(H3N1)	23	WVL	1	Breeders-broiler	4/05/2019	Pool of 5 cloacal swabs maximum
EPI_ISL_3914903	24–2-POO	A/Gallus gallus/Belgium/5228_0001/2019(H3N1)	24	WVL	2	Layers	20/05/2019	Pooled lung + trachea + intestine + brain
EPI_ISL_3914914	24–3-POO	A/Gallus gallus/Belgium/5228_0002/2019(H3N1)	24	WVL	3	Layers	20/05/2019	Pooled lung + trachea + intestine + brain
EPI_ISL_3914926	24–4-POO	A/Gallus gallus/Belgium/5228_0003/2019(H3N1)	24	WVL	4	Layers	20/05/2019	Pooled lung + trachea + intestine + brain
EPI_ISL_3914934	24–1-PCS	A/Gallus gallus/Belgium/4458_0001/2019(H3N1)	24	WVL	1	Layers	4/05/2019	Pool of 5 cloacal swabs maximum
EPI_ISL_3914938	26–1-POO	A/Gallus gallus/Belgium/4525_0001/2019(H3N1)	26	WVL	1	Layers	6/05/2019	Pooled lung + trachea + intestine
EPI_ISL_3914941	27–2-POO	A/Gallus gallus/Belgium/4759_0001/2019(H3N1)	27	WVL	2	Layers	10/05/2019	Pooled lung + trachea + intestine + brain
EPI_ISL_3914949	27–8-PTLB	A/Gallus gallus/Belgium/4538_0001/2019(H3N1)	27	WVL	8	Layers	6/05/2019	Pooled lung + trachea + intestine
EPI_ISL_3914953	28–2-PCS	A/Gallus gallus/Belgium/4492_0002/2019(H3N1)	28	OVL	2	Layers-outdoor	5/05/2019	Pool of 5 cloacal swabs maximum
EPI_ISL_3914955	29–2-PTS	A/Turkey/Belgium/4539_0001/2019(H3N1)	29	WVL	2	Broiler-turkey	5/05/2019	Pool of 5 tracheal swabs maximum

Accession nos.†	SeqID‡	Strain name	Outbreak no.	Province	Production unit	Production type	Sample date (dd/mm/yyyy)	Sample type
EPI_ISL_3914967	30–2-PCS	A/Gallus gallus/Belgium/5347_0002/2019(H3N1)	30	WVL	HOK 2	Layers	22/5/2019	Pool of 5 cloacal swabs maximum
EPI_ISL_3914978	30–1-PCS	A/Gallus gallus/Belgium/4543_0001/2019(H3N1)	30	WVL	1	Layers	6/05/2019	Pool of 5 cloacal swabs maximum
EPI_ISL_3914990	31–2-POO	A/Gallus gallus/Belgium/5055_0002/2019(H3N1)	31	WVL	2	Breeders-broiler	17/05/2019	Pooled lung + trachea + intestine + brain
EPI_ISL_3915000	31–3-POO	A/Gallus gallus/Belgium/5055_0003/2019(H3N1)	31	WVL	3	Breeders-broiler	17/05/2019	Pooled lung + trachea + intestine + brain
EPI_ISL_3915012	31-E-POO	A/Gallus gallus/Belgium/4633_0001/2019(H3N1)	31	WVL	HOK E	Breeders-broiler	8/05/2019	Pooled lung + trachea + intestine + brain
EPI_ISL_3915025	32–1-PCS	A/Gallus gallus/Belgium/4732_0002/2019(H3N1)	32	WVL	1	Layers-outdoor	9/05/2019	Pool of 5 cloacal swabs maximum
EPI_ISL_3915037	33–1-PCS	A/Gallus gallus/Belgium/4852_0001/2019(H3N1)	33	WVL	3	Breeders-layers	13/05/2019	Pool of 5 cloacal swabs maximum
EPI_ISL_3915049	33–4-PTS	A/Gallus gallus/Belgium/4753_0001/2019(H3N1)	33	WVL	4	Breeders-layers	10/05/2019	Pool of 5 tracheal swabs maximum
EPI_ISL_3915376	34–2-PCS	A/Gallus gallus/Belgium/4768_0002/2019(H3N1)	34	OVL	2	Layers	10/05/2019	Pool of 5 cloacal swabs maximum
EPI_ISL_3915377	35–4-PCS	A/Gallus gallus/Belgium/5063_0002/2019(H3N1)	35	WVL	4	Layers	16/5/2019	Pool of 5 cloacal swabs maximum
EPI_ISL_3915378	36–1-POO	A/Gallus gallus/Belgium/4846_0001/2019(H3N1)	36	WVL	1	Breeders-broiler	13/05/2019	Pooled lung + trachea + intestine + brain
EPI_ISL_3915379	36–2-POO	A/Gallus gallus/Belgium/4846_0002/2019(H3N1)	36	WVL	2	Breeders-broiler	13/05/2019	Pooled lung + trachea + intestine + brain
EPI_ISL_3915380	37-A-POO	A/Gallus gallus/Belgium/4890_0001/2019(H3N1)	37	WVL	a	Layers	14/052019	Pooled lung + trachea + intestine + brain
EPI_ISL_3915381	37-B-POO	A/Gallus gallus/Belgium/4890_0002/2019(H3N1)	37	WVL	b	Layers	14/052019	Pooled lung + trachea + intestine + brain
EPI_ISL_3915382	38–1-POO	A/turkey/Belgium/4914_0001/2019(H3N1)	38	WVL	1	Broiler-turkey	15/05/2019	Pooled lung + trachea +intestine + brain
EPI_ISL_3915383	39–2-PCS	A/Gallus gallus/Belgium/4982_0002/2019(H3N1)	39	OVL	2	Layers	14/05/2019	Pool of 5 cloacal swabs maximum
EPI_ISL_3915384	39–1-PCS	A/Gallus gallus/Belgium/5192_0002/2019(H3N1)	39	OVL	1	Layers	20/05/2019	Pool of 5 cloacal swabs maximum
EPI_ISL_3915385	40–1-PCS	A/Gallus gallus/Belgium/5060_0002/2019(H3N1)	40	WVL	1	Layers	16/05/2019	Pool of 5 cloacal swabs maximum
EPI_ISL_3915386	42–3-POO	A/Gallus gallus/Belgium/5051_0003/2019(H3N1)	42	WVL	3	Breeders-broiler	17/05/2019	Pooled lung + trachea + intestine + brain
EPI_ISL_3915387	44–1-PTL	A/Ostrich/Belgium/5049_0001/2019(H3N1)	44	WVL	1	Ostrich	5/06/2019	Pooled lung +trachea
EPI_ISL_3915388	45–1-POO	A/Gallus gallus/Belgium/5110_0001/2019(H3N1)	45	WVL	1	Breeders-broiler	17/05/2019	Pooled organs
EPI_ISL_3915389	45–3-POO	A/Gallus gallus/Belgium/5113_0001/2019(H3N1)	45	WVL	3	Breeders-broiler	17/05/2019	Pooled organs
EPI_ISL_3915390	45–2-POO	A/Gallus gallus/Belgium/5481_0002/2019(H3N1)	45	WVL	2	Breeders-broiler	24/05/2019	Pooled lung + trachea + intestine + brain
EPI_ISL_3915391	47–1-POO	A/Gallus gallus/Belgium/5593_0001/2019(H3N1)	47	WVL	1	Breeders-broiler	27/05/2019	Pooled lung + trachea + intestine + brain

Accession nos.†	SeqID‡	Strain name	Outbreak no.	Province	Production unit	Production type	Sample date (dd/mm/yyyy)	Sample type
EPI_ISL_3915392	48–1-POO	A/Gallus gallus/Belgium/5426_0001/2019(H3N1)	48	WVL	2	Breeders-layers	22/05/2019	Pooled lung + trachea + intestine + brain
EPI_ISL_3915393	49–4-PTS	A/Turkey/Belgium/5427_0001/2019(H3N1)	49	WVL	4	Broiler-turkey	21/05/2019	Pool of 5 tracheal swabs maximum
EPI_ISL_3915394	50–1-PCS	A/Gallus gallus/Belgium/5430_0002/2019(H3N1)	50	OVL	1	Layers	21/05/2019	Pool of 5 cloacal swabs maximum
EPI_ISL_3915395	50–2-PCS	A/Gallus gallus/Belgium/5432_0002/2019(H3N1)	50	OVL	2	Layers	21/05/2019	Pool of 5 cloacal swabs maximum
EPI_ISL_3915396	51–2-POO	A/Gallus gallus/Belgium/5484_0001/2019(H3N1)	51	WVL	2	Breeders-broiler	26/05/2019	Pooled organs
EPI_ISL_3915397	52–2-POO	A/Turkey/Belgium/5478_0001/2019(H3N1)	52	WVL	2	Broiler-turkey	24/05/2019	Pooled lung + trachea + intestine + brain
EPI_ISL_3915398	54–1-PO0	A/Gallus gallus/Belgium/5534_0001/2019(H3N1)	54	WVL	1	Layers	26/05/2019	Pooled lung + trachea + intestine + brain
EPI_ISL_3915516	56–7-POO	A/Gallus gallus/Belgium/5772_0001/2019(H3N1)	56	WVL	7	Breeders-broiler	3/06/2019	Pooled lung + trachea + intestine + brain
EPI_ISL_3915517	56–8-POO	A/Gallus gallus/Belgium/5772_0002/2019(H3N1)	56	WVL	8	Breeders-broiler	3/06/2019	Pooled lung + trachea + intestine + brain
EPI_ISL_3915399	56–1-POO	A/Gallus gallus/Belgium/5570_0001/2019(H3N1)	56	WVL	1	Breeders-broiler	27/05/2019	Pooled organs
EPI_ISL_3915400	57–14-iPCS	A/Gallus gallus/Belgium/5679_0002/2019(H3N1)	57	WVL	14	Breeders-broiler	29/05/2019	Isolate (pool of 5 cloacal swabs maximum)
EPI_ISL_3915401	57–11-PCS	A/Gallus gallus/Belgium/5869_0002/2019(H3N1)	57	WVL	11	Breeders-broiler	6/06/2019	Pool of 5 cloacal swabs maximum
EPI_ISL_3915402	58–1-PCS	A/Gallus gallus/Belgium/5680_0002/2019(H3N1)	58	WVL	1	Layers	29/05/2019	Pool of 5 cloacal swabs maximum
EPI_ISL_3915403	60–1-POO	A/Gallus gallus/Belgium/5769_0001/2019(H3N1)	60	WVL	1	Breeders-broiler	4/06/2019	Pooled lung + trachea + intestine + brain
EPI_ISL_3915404	62–1-POO	A/Gallus gallus/Belgium/5771_0001/2019(H3N1)	62	WVL	1	Layers	3/06/2019	Pooled organs
EPI_ISL_3915405	63–1-POO	A/Gallus gallus/Belgium/5770_0001/2019(H3N1)	63	OVL	1	Breeders - layer	3/06/2019	Pooled organs
EPI_ISL_3915406	64–1-PCS	A/Gallus gallus/Belgium/5766_0001/2019(H3N1)	64	OVL	2	Breeders-broiler	3/06/2019	Pool of 5 cloacal swabs maximum
EPI_ISL_3915407	65–2-PCS	A/Gallus gallus/Belgium/5819_0002/2019(H3N1)	65	WVL	2	Layers	1/06/2019	Pool of 5 cloacal swabs maximum
EPI_ISL_3915408	66–1-PCS	A/Gallus gallus/Belgium/5826_0002/2019(H3N1)	66	WVL	1	Layers	4/06/2019	Pool of 5 cloacal swabs maximum
EPI_ISL_3915409	66–2-PCS	A/Gallus gallus/Belgium/6121_0002/2019(H3N1)	66	WVL	2	Layers	13/06/2019	Pool of 5 cloacal swabs maximum
EPI_ISL_3915410	67–4-PTS	A/Gallus gallus/Belgium/5824_0001/2019(H3N1)	67	WVL	4	Breeders-broiler	4/06/2019	Pool of 5 tracheal swabs maximum
EPI_ISL_3915411	68–1-PCS	A/Gallus gallus/Belgium/5924_0002/2019(H3N1)	68	LUX	1	Layers	4/06/2019	Pool of 5 cloacal swabs maximum
EPI_ISL_3915412	69–2-POO	A/Gallus gallus/Belgium/5870_0002/2019(H3N1)	69	WVL	2	Broiler	6/06/2019	Pooled lung + trachea + intestine + brain
EPI_ISL_3915413	70–1-PTS	A/Gallus gallus/Belgium/5934_0001/2019(H3N1)	70	WVL	1	Layers	6/06/2019	Pool of 3 tracheal swabs maximum

Accession nos.†	SeqID‡	Strain name	Outbreak no.	Province	Production unit	Production type	Sample date (dd/mm/yyyy)	Sample type
EPI_ISL_3915414	71–6-PCS	A/Gallus gallus/Belgium/5956_0002/2019(H3N1)	71	WVL	6	Breeders-broiler	7/06/2019	Pool of 5 cloacal swabs maximum
EPI_ISL_3915415	71–7-PCS	A/Gallus gallus/Belgium/6132_0002/2019(H3N1)	71	WVL	7	Breeders-broiler	12/06/2019	Pool of 5 cloacal swabs maximum
EPI_ISL_3915416	72–1-iPOO	A/Gallus gallus/6079/2019(H3N1)	72	WVL	1	Breeders-broiler	12/06/2019	Isolate (pooled lung + trachea + intestine + brain)
EPI_ISL_3915417	73–1-PTS	A/Gallus gallus/Belgium/6082_0002/2019(H3N1)	73	WVL	1	Breeders-layer + quail	12/06/2019	Pool of 5 tracheal swabs maximum
EPI_ISL_3915418	73–2-PCS	A/Gallus gallus/Belgium/6094_0001/2019(H3N1)	73	WVL	2	Breeders-layer + quail	13/06/2019	Pool of 5 cloacal swabs maximum
EPI_ISL_3915419	75–1-iPCS	A/Gallus gallus/Belgium/6486/2019(H3N1)	75	WVL	1	Layers	22/06/2019	Isolate (pool of 5 cloacal swabs maximum)
EPI_ISL_3915420	77–1-iPOO	A/Gallus gallus/Belgium/6519/2019(H3N1)	77	WVL	1	Broilers	24/06/2019	Isolate (pooled lung + trachea + intestine + brain)
EPI_ISL_3915421	78–1-PCS	A/Gallus gallus/Belgium/6648_0001/2019(H3N1)	78	WVL	1	Breeders-broiler	28/06/2019	Pool of 5 cloacal swabs maximum
EPI_ISL_3915422	80–3-PCS	A/Gallus gallus/Belgium/7200_0001/2019(H3N1)	80	WVL	3	Breeders-layer	11/07/2019	Pool of 5 cloacal swabs maximum
EPI_ISL_3915423	81–1-iPOO	A/Gallus gallus/Belgium/6986/2019(H3N1)	81	WVL	1	Breeders-broiler	9/07/2019	Isolate (pooled lung + trachea + intestine + brain)

*LUX, Luxembourg; OVL, Oost-Vlaanderen; WVL, West-Vlaanderen.
†GISAID EpiFlu (https://www.gisaid.org) or Genbank accession numbers.
‡Sequence identification numbers from phylogeographic analysis.

Appendix Table 3. Sequencing coverage breadth for different avian influenza virus gene segments in study combining phylogeographic analyses and epidemiologic contact tracing to characterize atypically pathogenic H3N1 avian influenza epidemic, Belgium, 2019*

SeqID†	S1-PB2	S2-PB1	S3-PA	S4-HA	S5-NP	S6-NA	S7-MP	S8-NS
1–1-iPTL	100	100	100	100	100	100	100	100
0–1-PTL	100	100	0	100	100	92	100	100
2–1-iPTL	100	100	100	100	100	100	100	100
2–1-PTL-2	100	97	100	100	100	100	100	100
3–1-PTS	0	0	0	0	100	0	100	100
4–1-iPTL	100	100	100	100	100	100	100	100
4–1-PTL-2	0	0	0	100	0	0	0	100
5–1-PCS	100	100	100	100	100	100	100	100
6–1-iPTL	100	100	100	100	100	100	100	100
7–1-iBOW	100	100	100	100	100	100	100	100
8–1-PCS	100	100	100	100	100	100	100	100
8–3-PCS	100	100	100	100	100	100	100	100
9–2-BOW	100	100	100	100	100	100	100	100
11–1-PLT	100	100	100	100	100	100	100	100
11–1-POO	100	69	100	100	100	100	100	100
11–4-POO	82	100	49	100	100	100	100	100
12–1-iBOW	100	100	100	100	100	100	100	100
13–4-iBOW	100	100	100	100	100	100	100	100
13–5-BOW	61	100	100	100	100	100	100	100
14–1-PTL	100	100	100	100	100	100	100	100
14–1-iBOW	100	100	100	100	100	100	100	100
14–3-PTS-1	100	100	100	100	100	100	100	100
14–3-PTS-2	100	100	100	100	100	100	100	100
15–1-iBRA	100	100	100	100	100	100	100	100
16–1-POO	100	100	100	100	100	100	100	100
16–1-BOW	23	0	0	39	80	0	100	100
17–1-POO	0	0	0	87	96	0	94	100
19–2-PCS	92	85	58	0	99	82	100	100
19–1-PCS	100	100	100	100	100	100	100	100
20–1-PCS	100	100	100	100	100	100	100	100
21–3-PTL	100	100	100	100	100	100	100	100
21–1-POO	100	100	100	100	100	100	100	100
21–2-POO	100	100	100	100	100	100	100	100
22–6-PCS	100	100	100	100	100	100	100	100
22–4-PTS	100	100	100	100	100	100	100	100
23–2-PCS	100	100	100	100	100	100	100	100
23–1-PCS	100	100	100	100	100	100	100	100
24–2-POO	100	100	100	100	100	100	100	100
24–3-POO	100	100	100	100	100	100	100	100
24–4-POO	100	0	0	100	100	0	100	100
24–1-PCS	100	47	100	100	100	100	100	100
26–1-POO	0	0	0	0	100	0	100	100
27–2-POO	100	57	100	0	100	100	100	100
27–8-PTLB	42	0	100	100	100	0	100	100
28–2-PCS	100	14	31	100	100	0	100	100
29–2-PTS	100	100	100	100	100	100	100	100
30–2-PCS	100	100	100	100	100	100	100	100
30–1-PCS	100	100	100	100	100	100	100	100
31–2-POO	100	100	100	100	100	100	100	100
31–3-POO	100	100	100	100	100	100	100	100
31-E-POO	0	16	0	92	100	0	100	100
32–1-PCS	100	100	100	100	100	100	100	100
33–1-PCS	100	100	100	100	100	96	100	100
33–4-PTS	100	100	100	100	100	100	100	100
34–2-PCS	100	100	100	100	100	100	100	100
35–4-PCS	100	100	100	100	100	100	100	100
36–1-POO	35	0	0	0	0	0	85	100
36–2-POO	100	100	100	100	100	100	100	100
37-A-POO	100	100	100	100	100	100	100	100
37-B-POO	100	100	100	100	100	100	100	100
38–1-POO	100	0	0	100	100	48	100	100
39–2-PCS	100	100	100	100	100	100	100	100
39–1-PCS	100	100	100	100	100	100	100	100
40–1-PCS	0	29	26	100	0	100	100	100
42–3-POO	100	100	100	100	100	100	100	100
44–1-PTL	19	0	0	0	0	0	0	100

SeqID†	S1-PB2	S2-PB1	S3-PA	S4-HA	S5-NP	S6-NA	S7-MP	S8-NS
45–1-POO	100	100	0	0	0	0	100	100
45–3-POO	100	100	100	100	100	100	100	100
45–2-POO	100	100	100	100	100	100	100	100
47–1-POO	100	100	100	100	100	100	100	100
48–1-POO	100	100	100	100	100	100	100	100
49–4-PTS	100	100	100	100	100	100	100	100
50–1-PCS	100	100	100	100	100	100	100	100
50–2-PCS	100	100	100	100	100	100	100	100
51–2-POO	100	100	100	100	100	100	100	100
52–2-POO	23	0	19	0	100	0	100	59
54–1-PO0	70	93	0	93	100	93	100	100
56–7-POO	100	100	100	100	100	100	100	100
56–8-POO	100	100	100	100	100	100	100	100
56–1-POO	100	0	100	0	0	100	100	100
57–14-iPCS	100	100	100	100	100	100	100	100
57–11-PCS	0	0	0	70	100	100	100	100
58–1-PCS	100	100	100	100	100	100	100	100
60–1-POO	63	0	53	0	90	0	100	100
62–1-POO	100	100	100	100	100	100	100	100
63–1-POO	100	69	24	100	100	100	100	100
64–1-PCS	35	0	100	100	100	0	100	100
65–2-PCS	100	100	100	100	100	100	100	100
66–1-PCS	100	100	100	100	100	100	100	100
66–2-PCS	100	100	97	100	100	100	100	100
67–4-PTS	12	0	0	0	100	0	100	100
68–1-PCS	100	100	100	100	100	100	100	100
69–2-POO	100	100	99	100	100	100	100	100
70–1-PTS	100	100	100	100	100	100	100	100
71–6-PCS	0	52	38	100	100	0	100	100
71–7-PCS	100	100	100	100	100	100	100	100
72–1-iPOO	100	100	100	100	100	100	100	100
73–1-PTS	100	100	100	100	100	100	100	100
73–2-PCS	100	100	100	100	100	100	100	100
75–1-iPCS	100	100	100	100	100	100	100	100
77–1-iPOO	100	100	100	100	100	100	100	100
78–1-PCS	0	0	0	0	0	0	100	95
80–3-PCS	100	100	100	100	100	100	100	100
81–1-iPOO	100	100	100	100	100	100	100	100

*Values are percent sequencing coverage. S1-PB2, segment 1-polymerase basic protein 2; S2-PB1, segment 2-polymerase basic protein 1; S3-PA, segment 3-polymerase acidic protein; S4-HA, segment 4-hemagglutinin; S5-NP, segment 5-nucleoprotein; S6-NA, segment 6-neuraminidase; S7-MP, segment 7-matrix protein; S8-NS, segment 8-nonstructural protein.

†Sequence identification numbers from phylogeographic analysis.

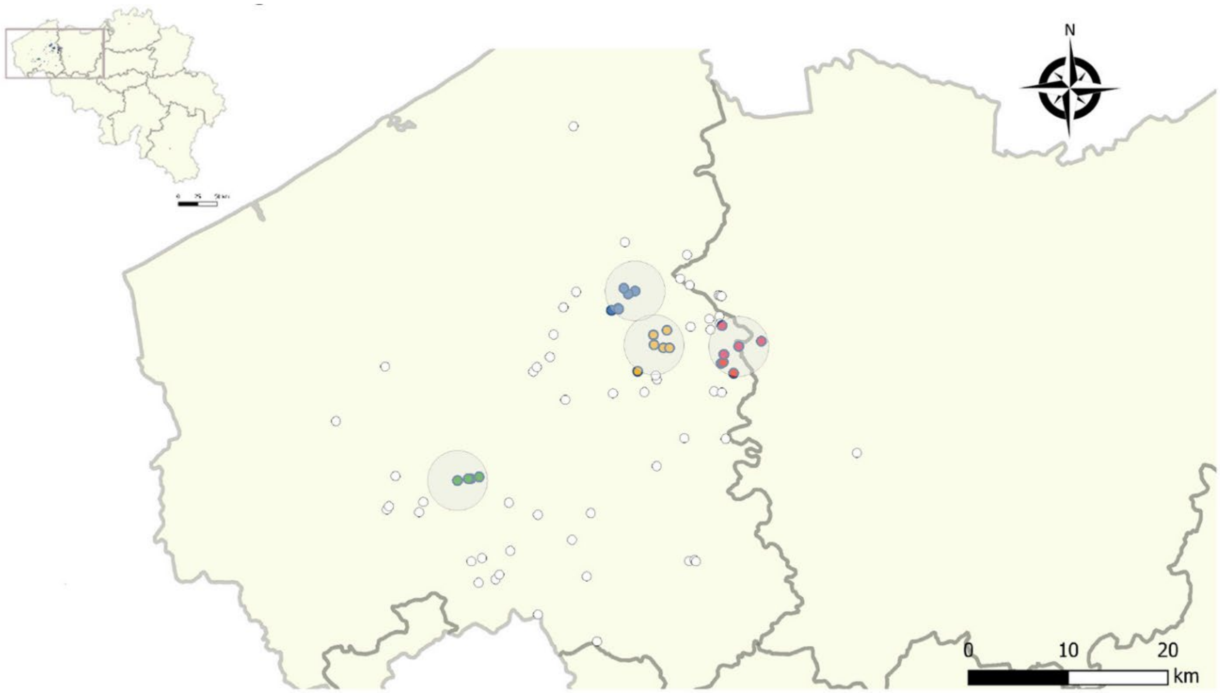

Appendix Figure 1. SaTScan spatiotemporal clustering of avian influenza H3N1–affected farms in study combining phylogeographic analyses and epidemiologic contact tracing to characterize atypically pathogenic H3N1 avian influenza epidemic, Belgium, 2019. We detected spatiotemporal case clusters by using SaTScan v.9.6 (https://www.SaTScan.org). Clusters with a 3 km radius are plotted on the map of West Flanders and East Flanders, Belgium, using colors to identify the order of occurrence. Cluster 1 included the index case (red), clusters 2 (yellow) and 3 (blue) represented short distance dispersal in a westerly direction, and cluster 4 (green) represented a medium distance (<50 km) dispersal in a southwesterly direction.

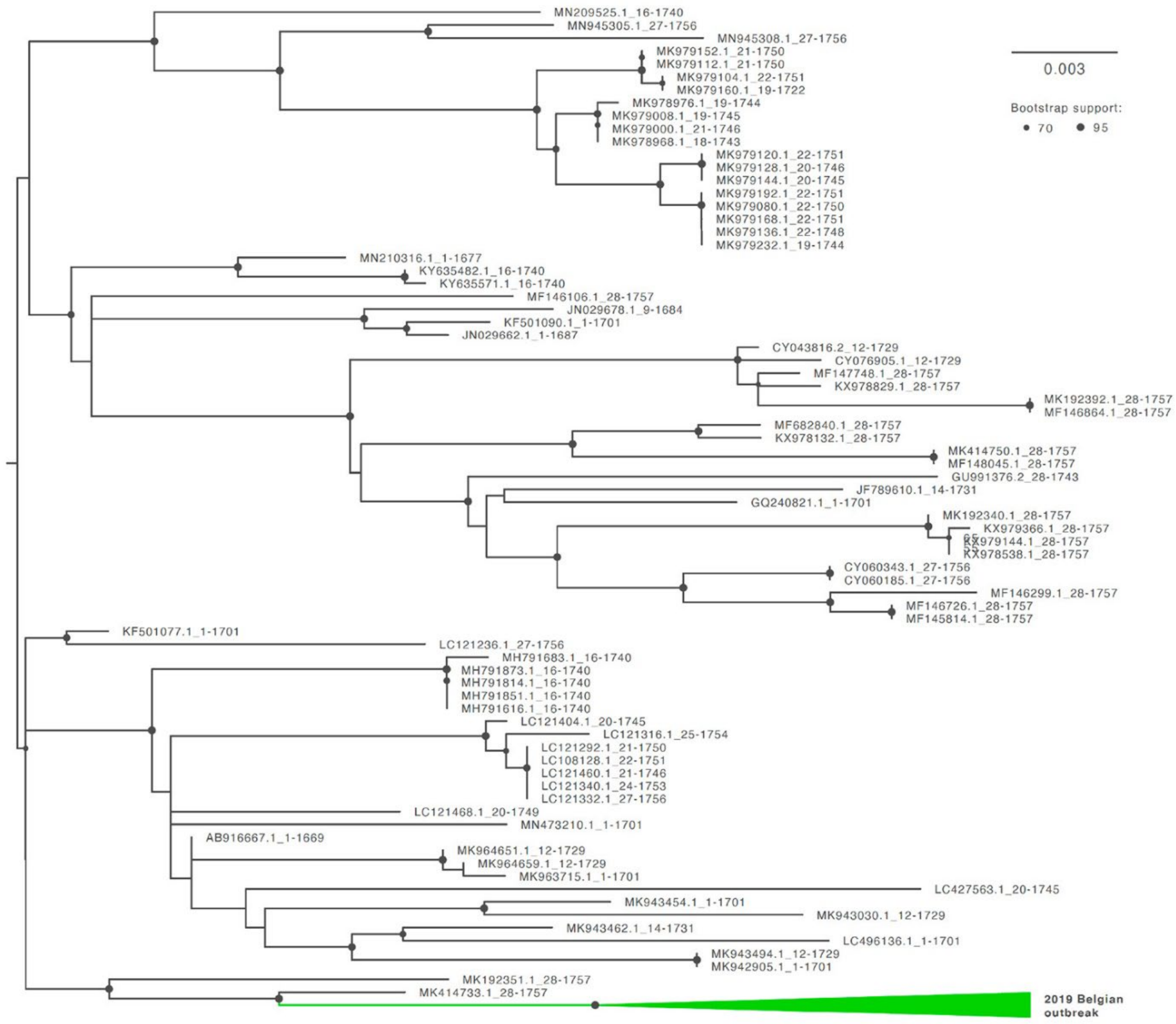

Appendix Figure 2. Preliminary phylogenetic analysis of the H3 gene segment in study combining phylogeographic analyses and epidemiologic contact tracing to characterize atypically pathogenic H3N1 avian influenza epidemic, Belgium, 2019. Phylogenetic tree was generated for the hemagglutinin 3 (H3) segment of avian inflluenza virus by using the maximum-likelihood method. Analysis was based on all hemagglutinin gene sequences generated in the present study and 80 H3Nx hemagglutinin gene segment sequences from outside of Belgium selected to represent the diversity of H3Nx viruses circulating in Eurasia before the introduction of the H3N1 virus in Belgium in 2019 (GenBank sequences available on February 12, 2020). Scale bar indicates nucleotide substitutions per site.

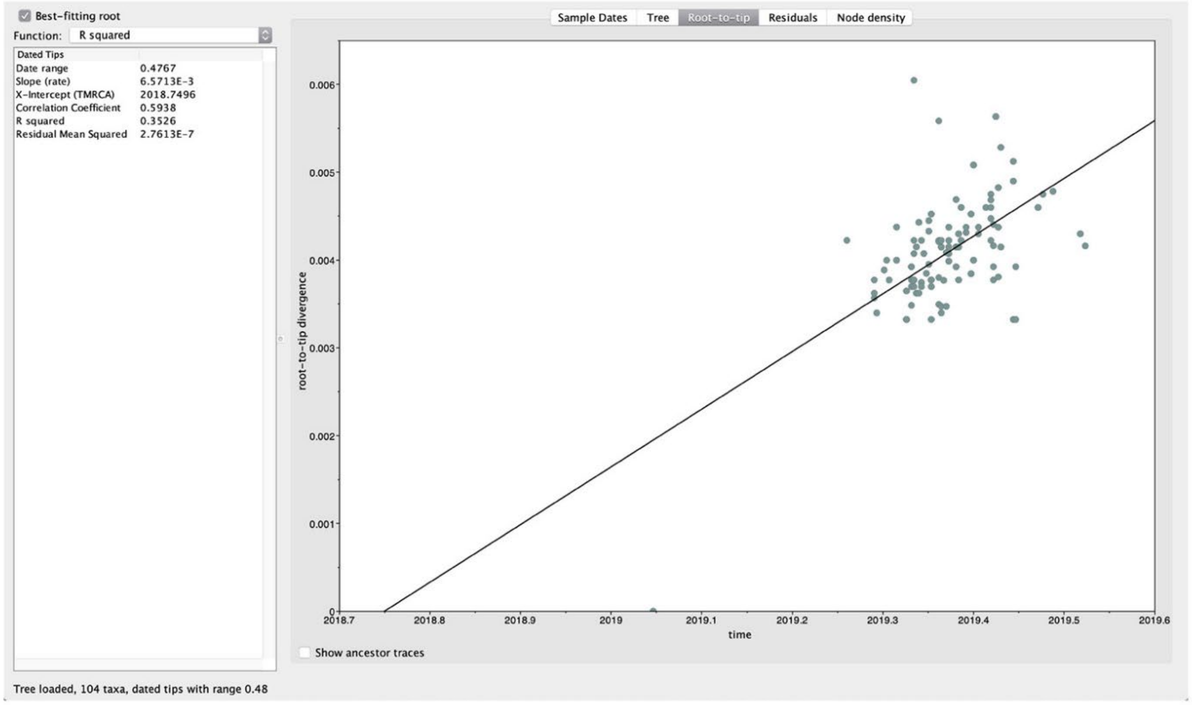

Appendix Figure 3. Root-to-tip regression analysis of phylogenetic temporal signal in study combining phylogeographic analyses and epidemiologic contact tracing to characterize atypically pathogenic H3N1 avian influenza epidemic, Belgium, 2019. We plotted root-to-tip genetic distances against sequence sampling times from July 2018 through June 2019 by using the program TempEst (*10*), best-fitting the root by maximizing the coefficient of determination R^2 (R^2 = 0.32).

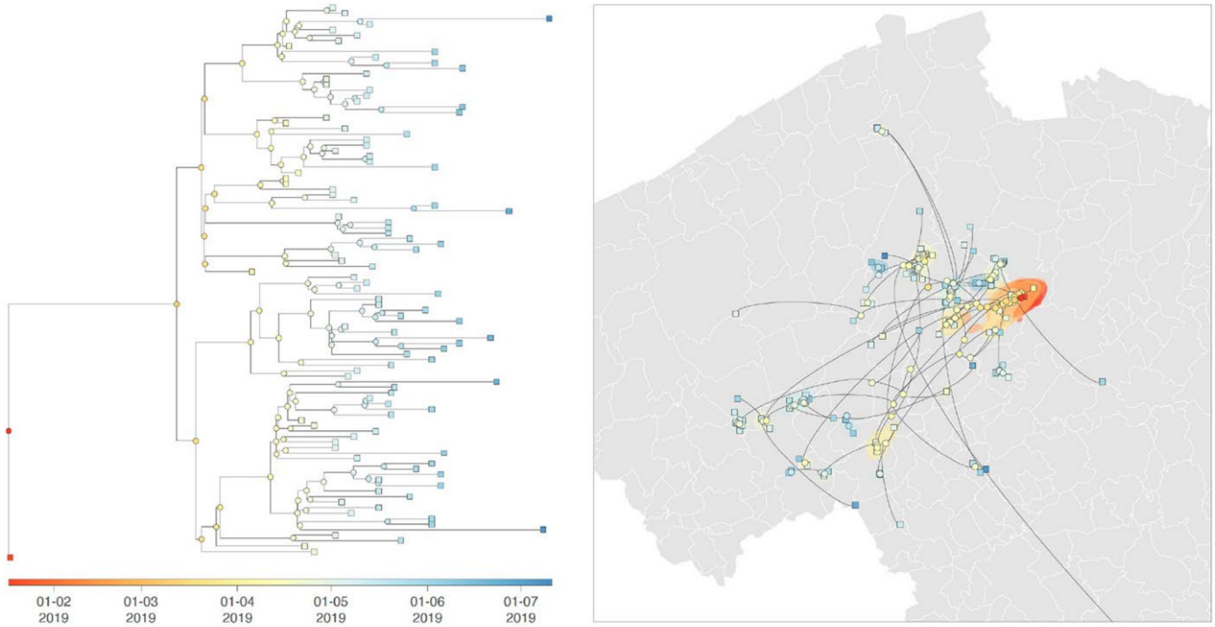

Appendix Figure 4. Phylogeographic reconstruction of the dispersal history of H3N1 lineages during the 2019 Belgian epidemic in study combining phylogeographic analyses and epidemiologic contact tracing to characterize atypically pathogenic H3N1 avian influenza epidemic, Belgium, 2019. Time-scaled maximum clade credibility (MCC) tree (left panel) was obtained by continuous phylogeographic inference and was based on 1,000 posterior trees. The MCC tree was superimposed on 80% highest posterior density polygons (shaded regions) reflecting phylogeographic uncertainty associated with the inferred position of internal nodes (right panel). Tips (squares) and internal nodes (circles) of the MCC tree are colored according to the outbreak date. Dispersal direction of viral lineages are indicated by the edge curvature (dispersal direction is anticlockwise).
